# Supplementary material for: Isolation of Artemisia capillaris membrane-bound di-prenyltransferase for phenylpropanoids and redesign of artepillin C in yeast
Source: Commun Biol. 2019 Oct 18;2:384. doi: 10.1038/s42003-019-0630-0 (PMC6802118; doi:10.1038/s42003-019-0630-0)
Supplement: Supplementary file 1 — Supplementary Information [file 42003_2019_630_MOESM1_ESM.pdf]

a

N75 length	587
N50 length	1,089
N25 length	1,837
Minimum length	249
Maximum length	13,155
Average length	848
Total bases	35,531,974
Contig counts	41,856
Unigene counts	39,615

b

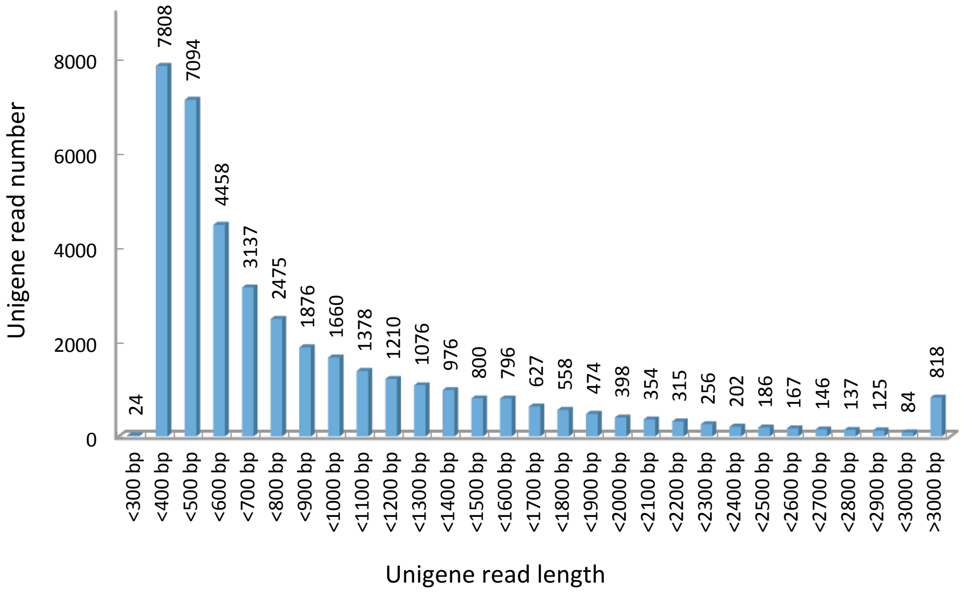

Supplementary Figure 1. RNA-seq data of *A. capillaris* leaves

a. Basic information for assembled contigs. A unigene set of 39,615 sequences was generated by TGICL clustering of 41,856 contigs.

b. Histogram of unigenes by sequence length.

Munakata *et al.*

a

A. capillaris unigene ID	Query with highest homology	Amino acid identity(%)	Predicted function
1147	HaCOX10	84.1	COX10
1403	LsVTE2-1	94.7	VTE2-1
4037	LsVTE2-1	70.2	VTE2-1
4523	HaATG4	90.0	ATG4
5921	HaABC4	75.8	ABC4
6131	HaVTE2-2	62.6	VTE2-2
6132	HaVTE2-2	61.3	VTE2-2
9515	LsVTE2-1	43.5	Unknown
11013	HaATG4	95.0	ATG4
11389	HaVTE2-2	84.6	VTE2-2
12392	HaVTE2-2	93.6	VTE2-2
14791	HaPPT	93.9	PPT
19105	HaCOX10	78.2	COX10
20817	LsVTE2-1	22.1	Unknown
21310	HaCOX10	66.6	COX10
22890	HaPPT	31.6	Unknown
22891	HaPPT	35.5	Unknown
27493	HaCOX10	31.1	Unknown

b

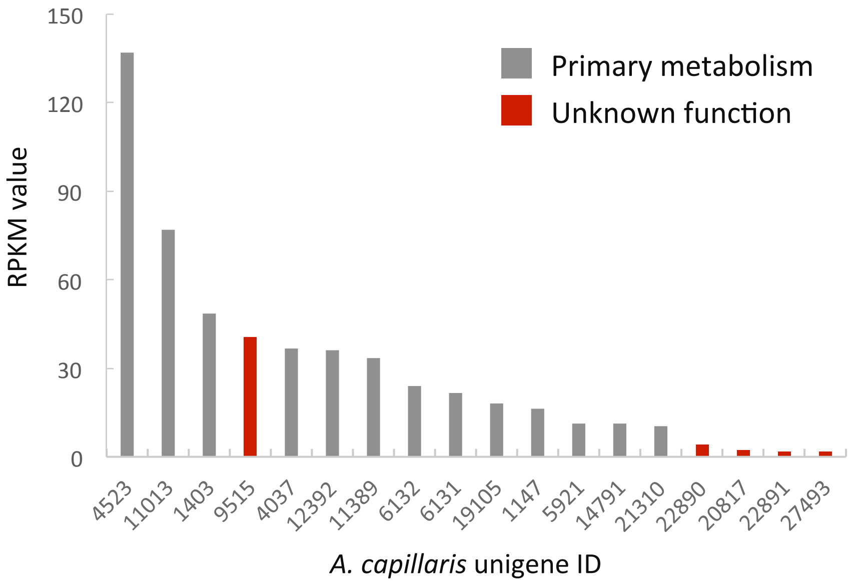

Supplementary Figure 2. ***In silico*** **analysis of the UbiA superfamily in** ***A. capillaris*** **RNA-seq unigenes**

a. Grouping of unigenes in the UbiA superfamily. Asteraceae proteins belonging to each of six UbiA subfamilies dedicated to primary metabolism (LsVTE2-1, HaVTE2-2, HaABC4, HaATG4, HaCOX10 and HaPPT) were selected as queries. Each unigene was functionally annotated according to the percent amino acid identity with the query protein and assorted relative to highest homology to the unigene. If amino acid identity was higher than 60%, the unigene was predicted to have the same function as the query. Unigenes showing amino acid identities lower than this threshold were selected as candidates possessing unknown functions. Unigene9515 contains the full CDS of *AcPT1*.

b. RPKM values of UbiA unigenes (n = 1). Unigenes predicted to be related to primary metabolism and to have unknown functions are highlighted in gray and red, respectively.

Munakata *et al.*

a

Predicted transit peptide

MASLTVGSLC KPTSNGLSIL VTSSSSLSTG AHASNFLRIS KVENNVSAQF 50

QRRGYKNHFG QSLHEPLSLQ KMDEEKFKLN AASTNNPQFD ATHDLVKPTE 100

SVISFLEVLF RFIRPYAAVG TVLCIASMSL LTVEKLSDFS PLFFMKVLQA 150

1st aspartate-rich motif

LVGAMFMQMW VCGI**NQICDI ELD**KINKPSL PLASGELSMT TAITVSALSA 200

IMSFSIGWIA SPALFWGFVG WFVVGTAYSA NLPWLRWKRF PLTSAFYMLC 250

2nd aspartate-rich motif

SRALVVPIGY YLHMQKSIHG GSALLSRPIL FAVGMLSAFC ISTIFF**KDIP** 300

DIEGDRMHGI KSLAITLGEK RTFWMCIWIL EIAYVAAAFF GATSPITWSK 350

YITVISHLAM ALALWTRAKS TDVKNKDAVQ SMYYFLWQLF FAEYGLIALV 400

R 401

b

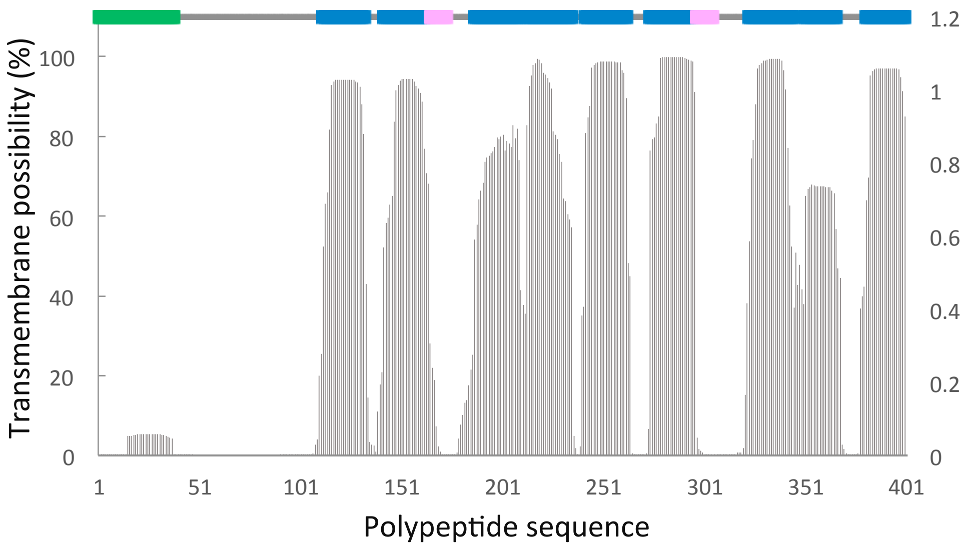

Supplementary Figure 3. ***In silico*** **analysis of the AcPT1 polypeptide**

a. Polypeptide sequence of AcPT1. The two aspartate-rich motifs and the transit peptide predicted by the ChloroP program are shown in pink and green boxes, respectively.

b. Transmembrane regions of AcPT1 predicted by the TMHMM program. Amino acids with high transmembrane possibilities (> 50%) are shown in blue as components of transmembrane regions, together with the aspartate-rich motifs (pink bars) and the predicted transit peptide (green bar).

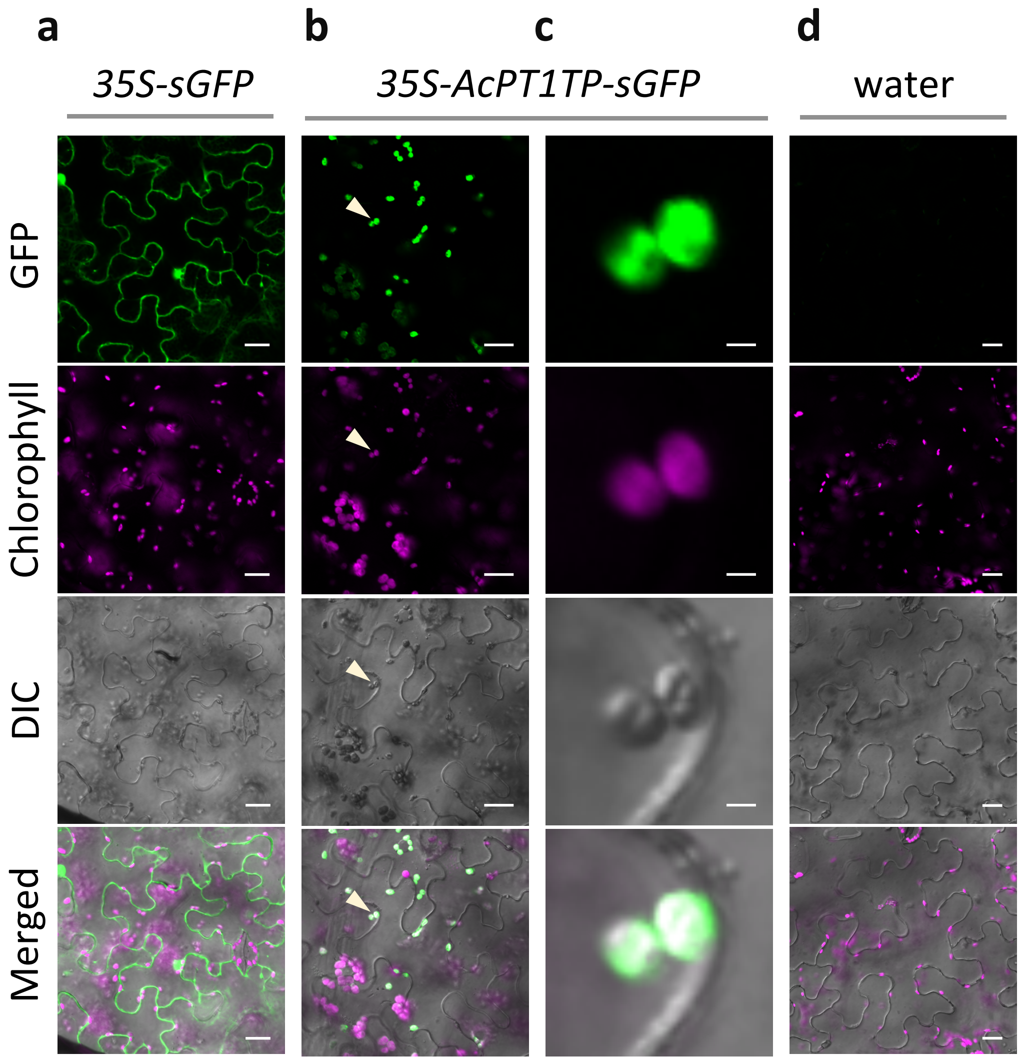

Supplementary Figure 4. Microscopic investigation of AcPT1TP-sGFP expressed in *N. benthamiana* epidermal cells

a – c. *sGFP* (**a**) and *AcPT1TP-sGFP* (**b** and **c**) were transiently expressed in epidermal cells of *N. benthamiana* leaves by agroinfiltration. **c**. Regions indicated by white arrowheads in **b** are enlarged.

d. Water was infiltrated into the leaves as a negative control. The images show GFP signaling, chlorophyll autofluorescence, DIC, and their merged image. For merging, contrast of images is unbiasedly adjusted with magenta used as a pseudo-color for the chlorophyll signal. Bars represent 20 µm (**a**, **b**, and **d**) and 2 µm (**c**).

Munakata *et al.*

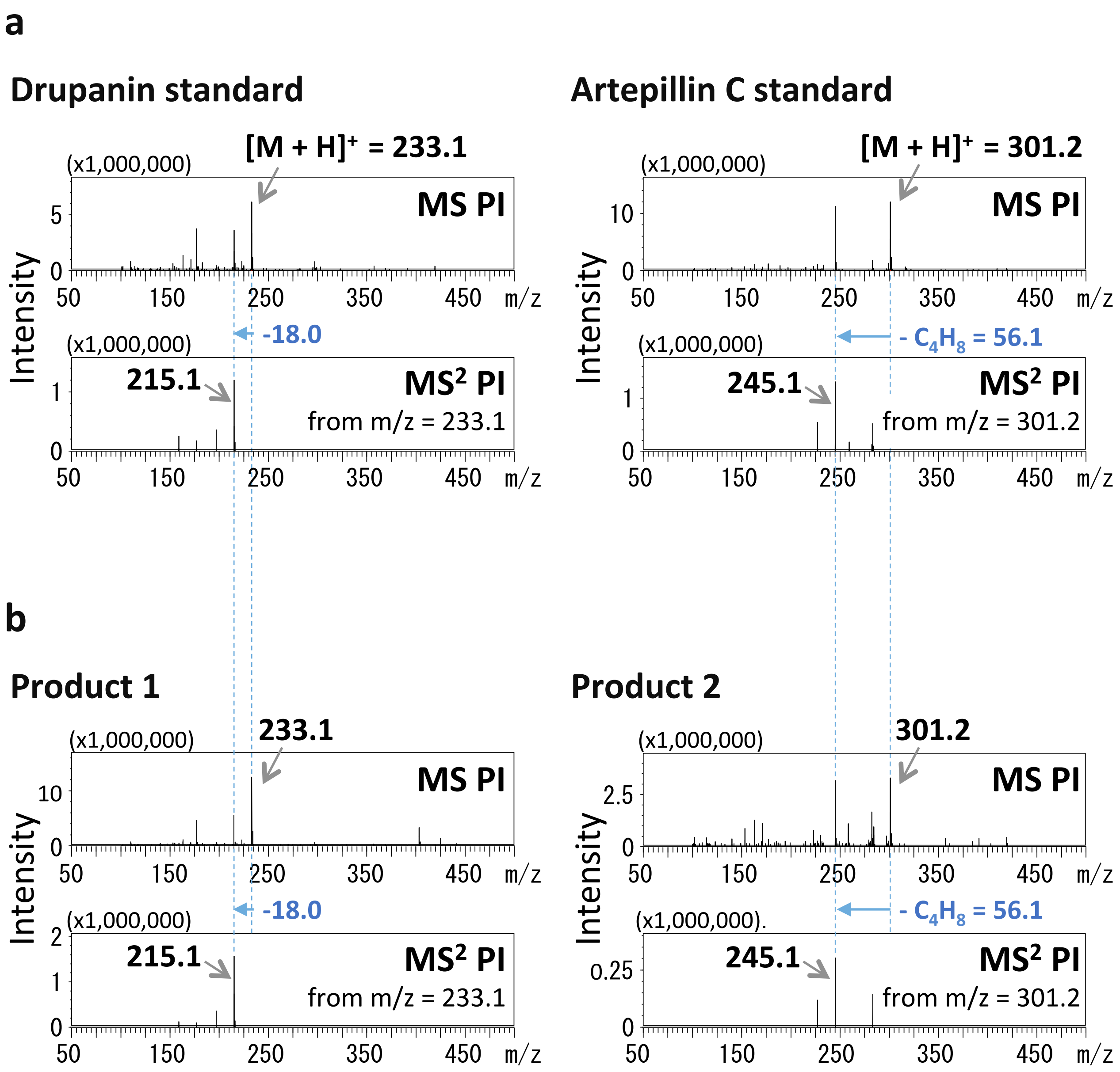

Supplementary Figure 5. MS-based identification of enzymatic reaction products in *p*-coumaric acid dimethylallyltransferase assay of AcPT1

MS and MS^2 spectra of standard specimens (**a**) and reaction products (**b**) detected in the positive ion mode.

a

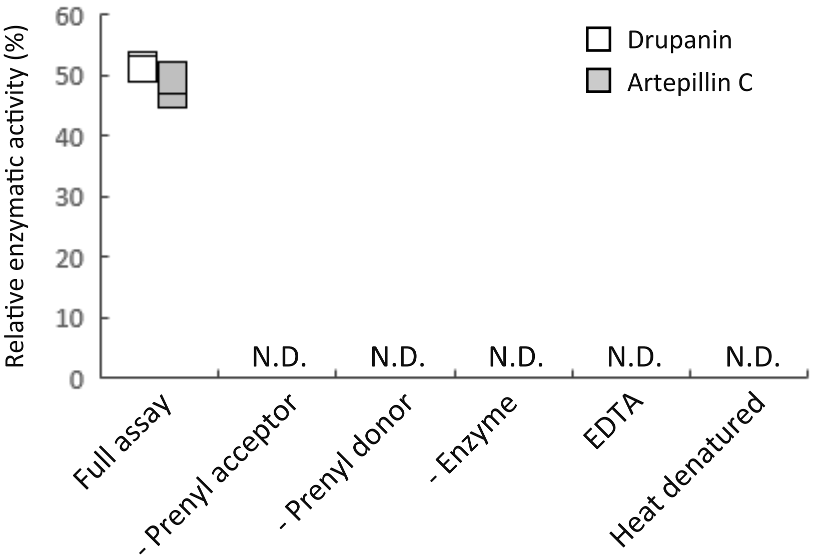

b

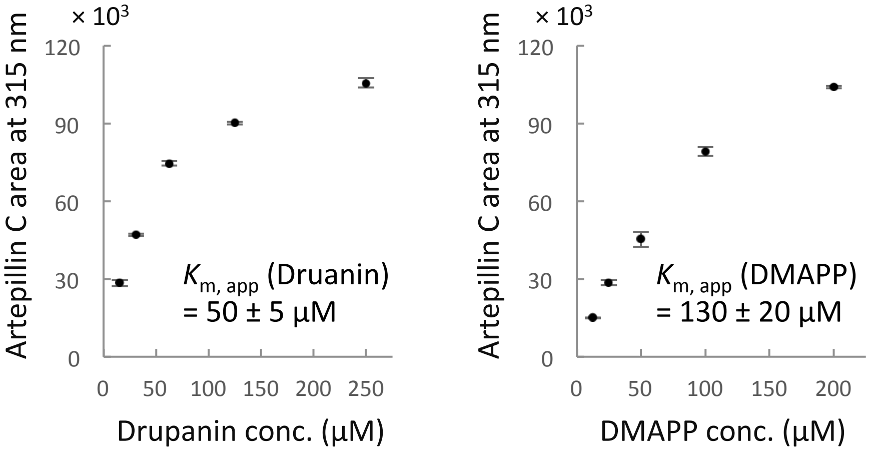

Supplementary Figure 6. Detailed enzymatic properties of AcPT1

a. Negative control reactions for the *p*-coumaric acid dimethylallyltransferase activity of AcPT1. Shown are overnight reactions without *p*-coumaric acid (- prenyl acceptor), DMAPP (- prenyl donor), or microsomes (- enzyme), and the addition of EDTA instead of $MgCl_2$ (EDTA) and the use of heat-denatured enzyme (Heat denatured) were also tested. The sum of the averages of produced drupanin and artepillin C in the full assay condition was set at 100%. Values (n = 3 independent experiments) are shown as box plots (center line, median; box limits, first and third quartiles or minimum and maximum).

b. Kinetic analysis of AcPT1 using drupanin and DMAPP as substrate pair. The reaction time was shortened to 20 min to calculate apparent K_m values for drupanin and DMAPP. Values are means ± standard errors (n = 3 independent experiments).

Munakata *et al.*

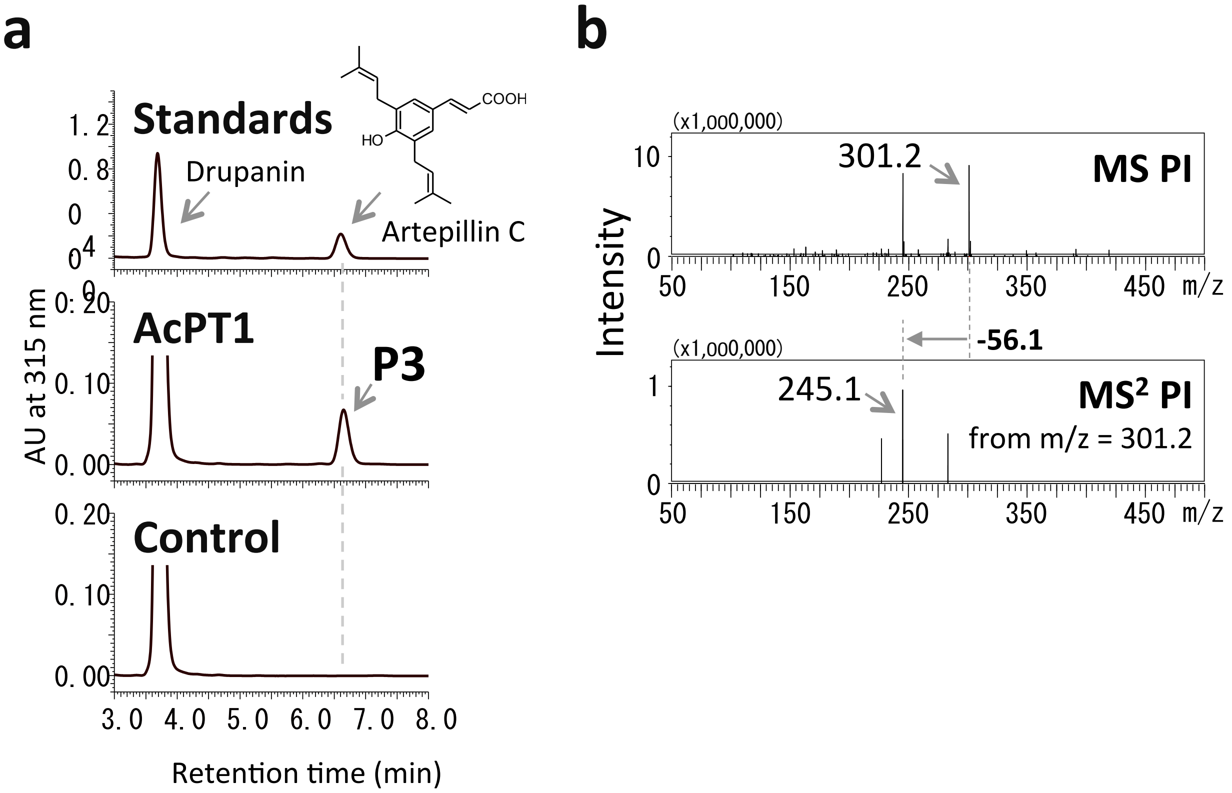

Supplementary Figure 7. LC/MS analysis of enzymatic reaction mixtures of AcPT1 in substrate specificity test

a and **b.** UV chromatogram of a drupanin dimethylallyltransferase reaction mixture with AcPT1 (**a**), and MS and MS^2 spectra of this reaction product, P3 (**b**).

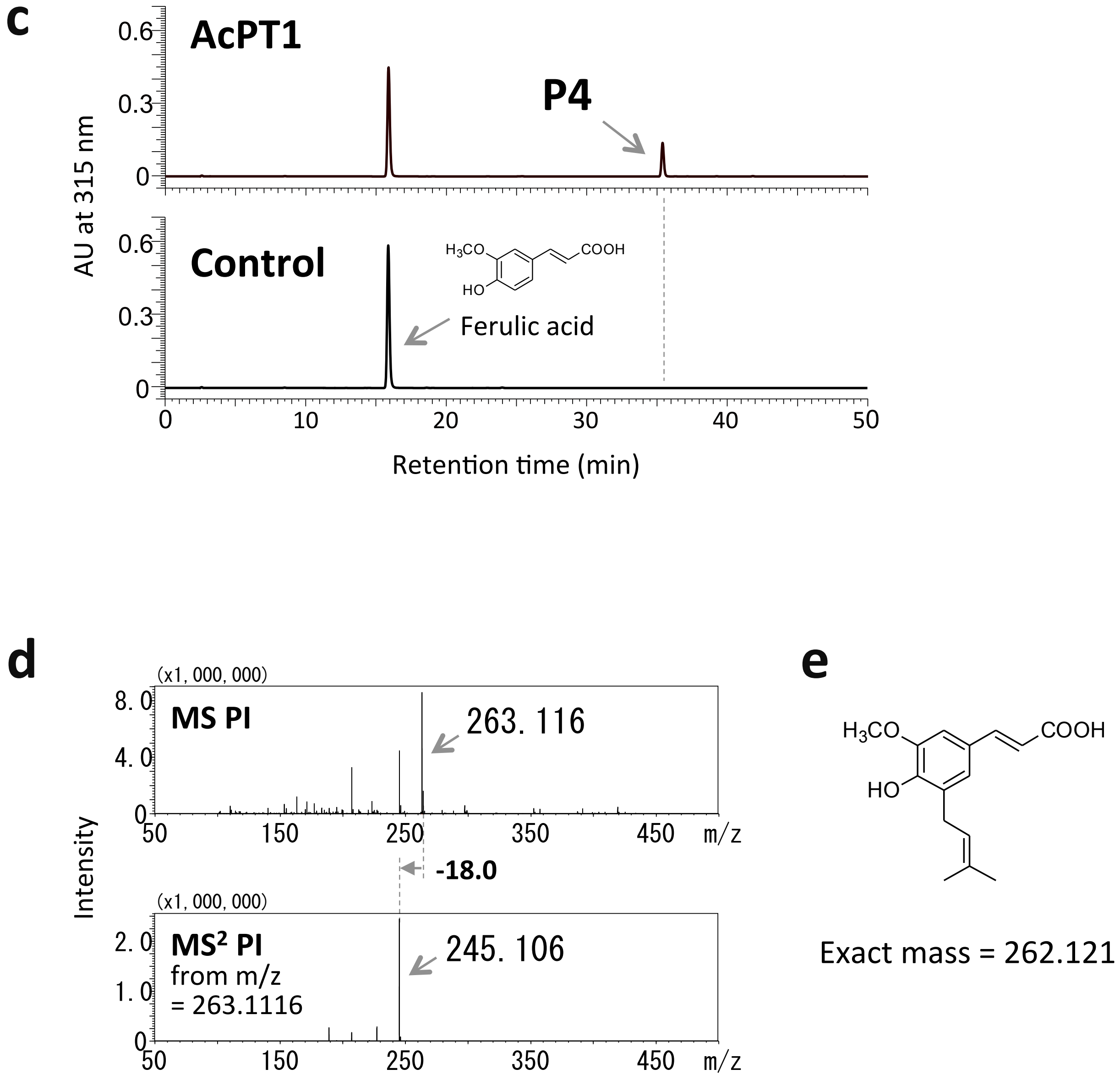

Supplementary Figure 7. LC/MS analysis of enzymatic reaction mixtures of AcPT1 in substrate specificity test ***- continued***

c - e. UV chromatogram of ferulic acid dimethylallyltransferase reaction mixture with AcPT1 (**c**), MS and MS^2 spectra of this reaction product, P4 (d), and its predicted chemical structure (**e**).

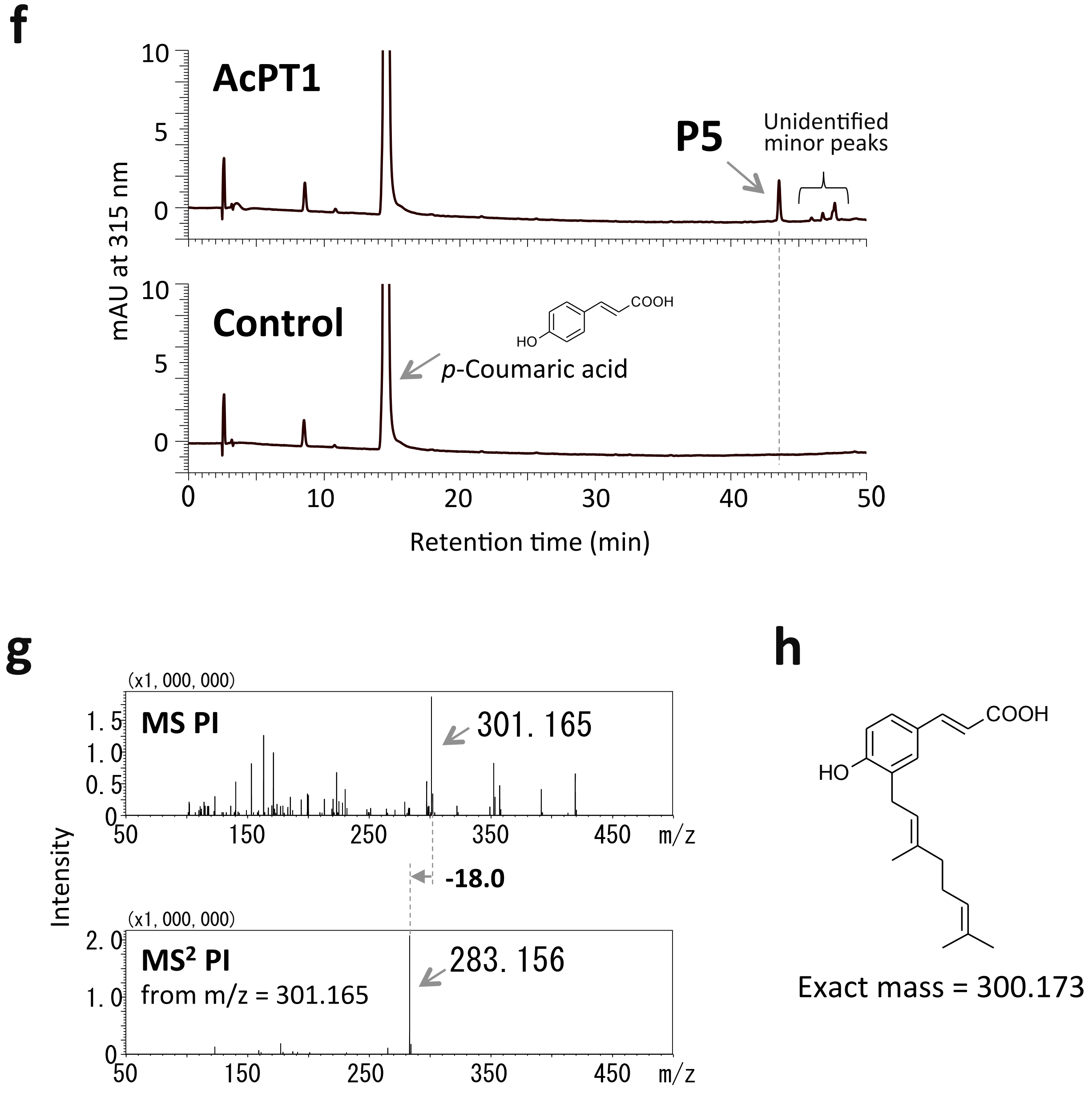

Supplementary Figure 7. LC/MS analysis of enzymatic reaction mixtures of AcPT1 in substrate specificity test - ***continued***

f - h. UV chromatogram of *p*-coumaric acid geranyltransferae reaction mixture with AcPT1 (**f**), MS and MS^2 spectra of the reaction product 5 (**g**), and its predicted chemical structure (**h**).

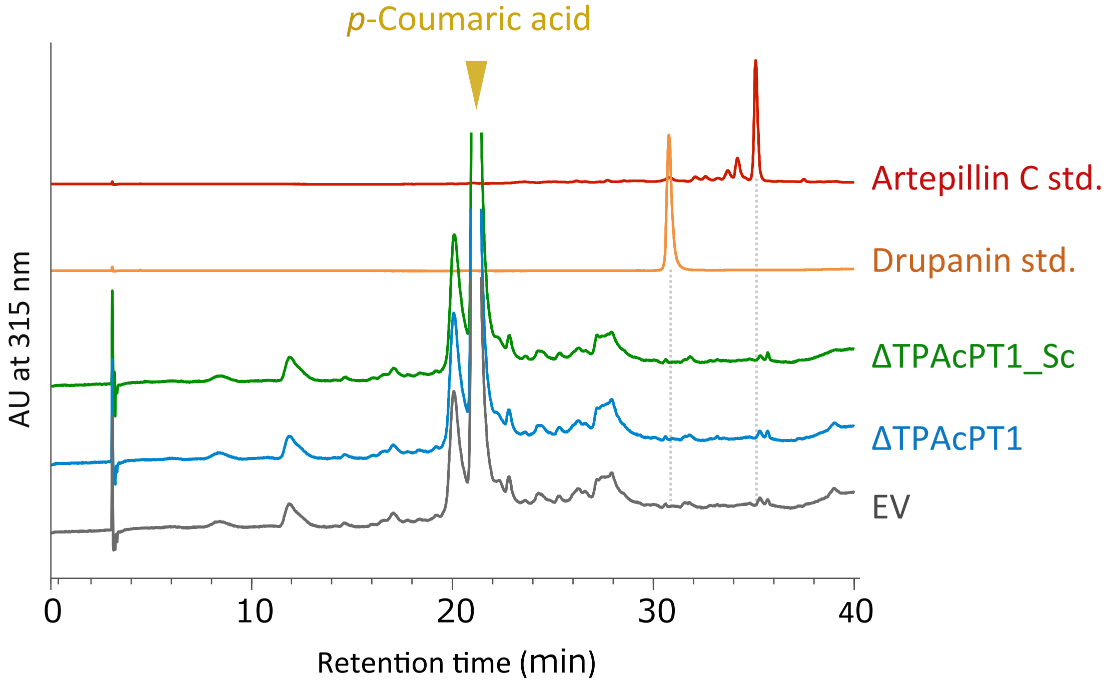

Supplementary Figure 8. UV chromatograms of medium fractions from the ΔTPAcPT1 transformants of the yeast COUM11 strain

Plasmids containing *ΔTPAcPT1* and *ΔTPAcPT1_Sc* were introduced into the yeast COUM11 strain engineered to produce *p*-coumaric acid by introduction of *PAL*, *C4H*, and *CPR*. Twenty-four hours after galactose treatment to induce expression of all foreign genes, phenolic compounds were extracted from the media of these yeast cultures and analyzed by HPLC.

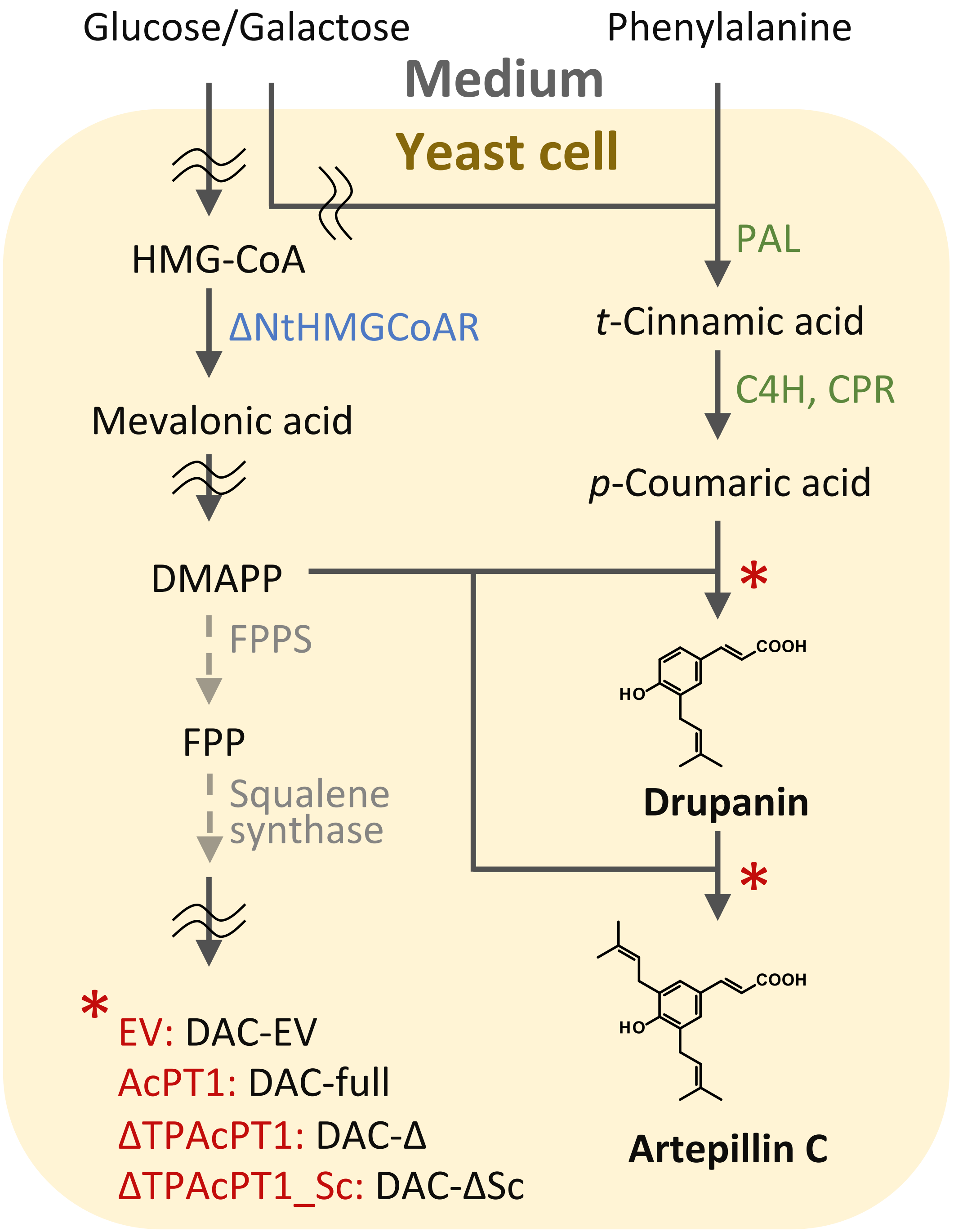

Supplementary Figure 9. Metabolic design of DAC yeast strains
The DD104 strain with a large DMAPP pool due to mutations in endogenous *FPPS* and *squalene synthase* was used as an expression host. *Truncated HMGCoA Reductase* (*ΔNtHMGCoAR*), *PAL*, *C4H*, and *CPR* were introduced into DD104 to increase quantities of AcPT1 substrates. The transformant was further genetically modified by pESC-His-EV, pESC-His-*AcPT1*, pESC-His-*ΔTPAcPT1* (a truncated CDS lacking its transit peptide), and pESC-His-*ΔTPAcPT1_Sc* (a codon-optimized *ΔTPAcPT1* for expression in budding yeast cells) to establish the DAC-EV, DAC-full, DAC-Δ, and DAC-ΔSc strains, respectively. All the transgenes were expressed under the control of galactose-inducible promoters.

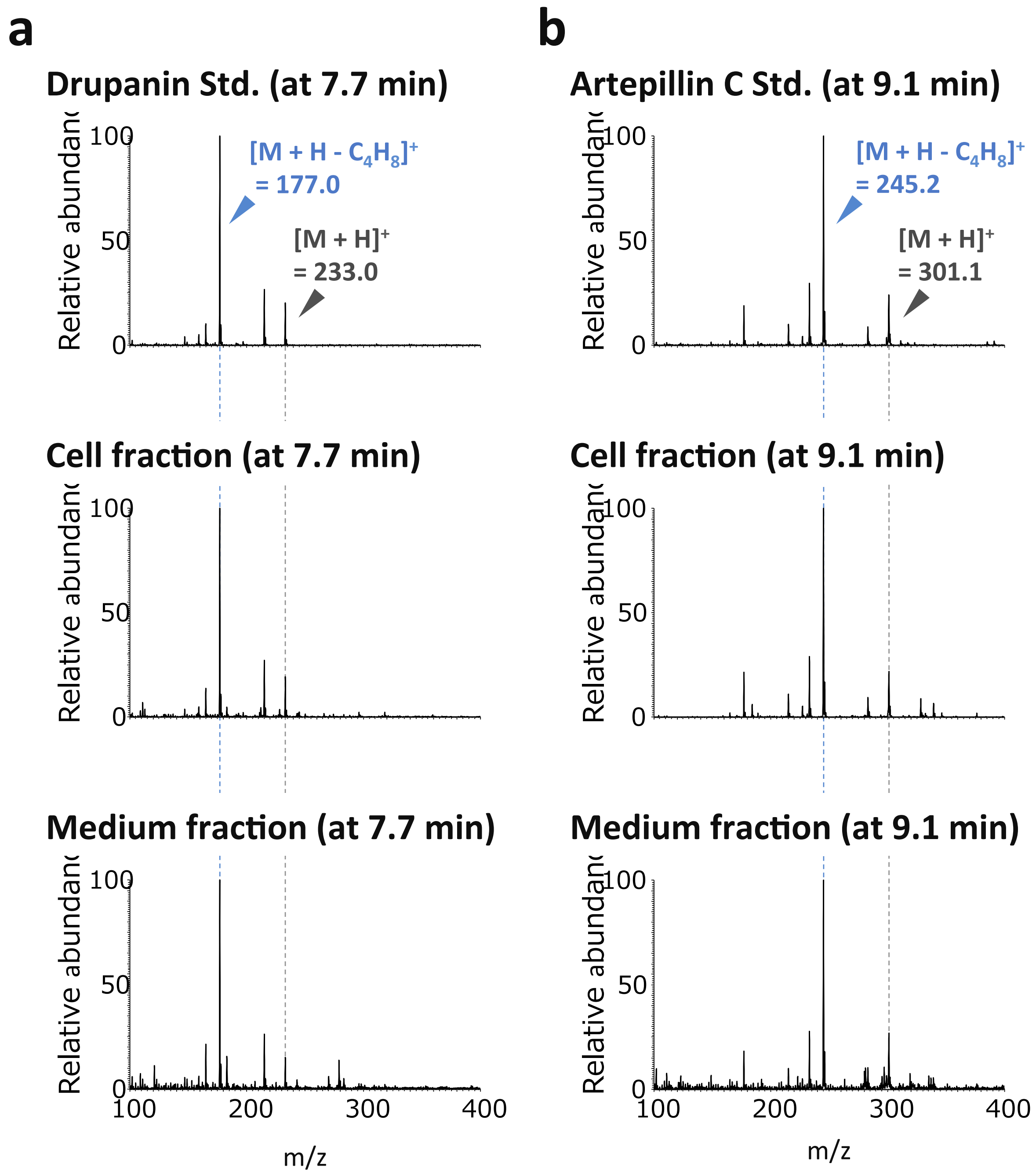

Supplementary Figure 10. MS spectra of *p*-coumarate derivatives from the DAC-ΔSc strain
MS spectra at retention times of 7.7 min (**a**) and 9.1 min (**b**) on MS chromatograms of standard specimens of drupanin and artepillin C, and extracts from the DAC-ΔSc culture.

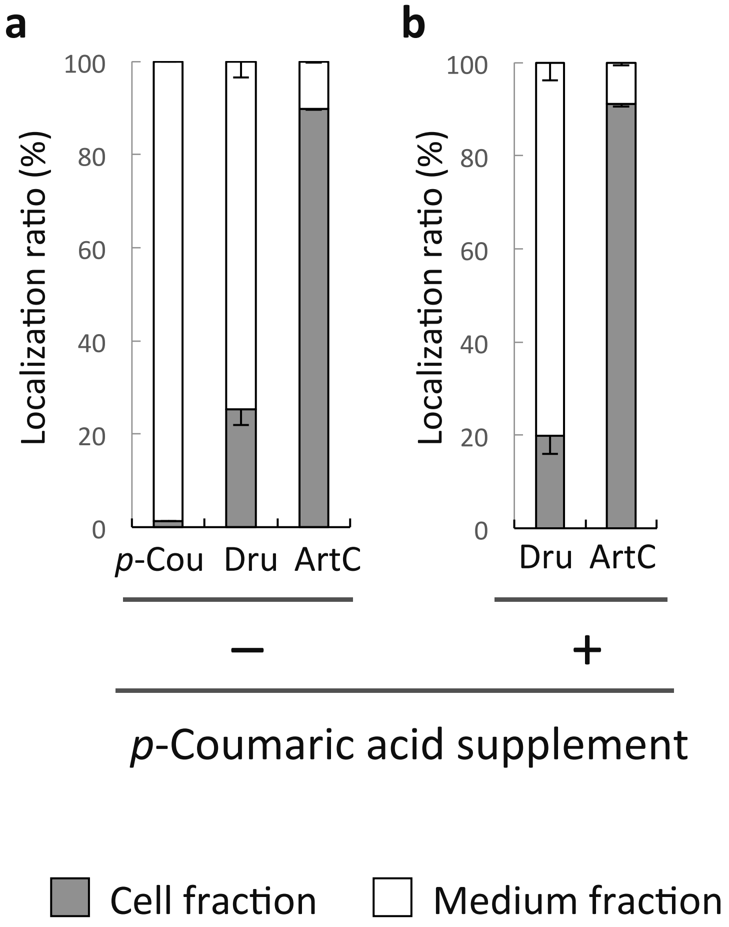

Supplementary Figure 11. Proportion of produced phenylpropane derivatives in DAC-ΔSc cultures
Proportion of *p*-coumaric acid (only for **a**), drupanin and artepillin C in cell and medium fractions of DAC-ΔSc cultures without (**a**) and with (**b**) *p*-coumaric acid administration. Values are means ± standard errors (n = 3 independent experiments).

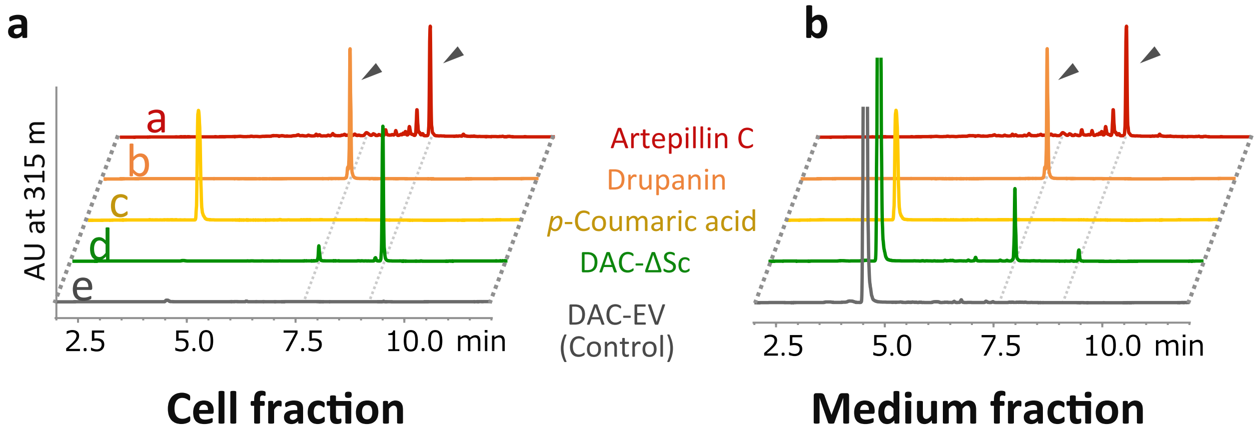

Supplementary Figure 12. UV chromatograms of extracts of the DAC-ΔSc strain supplemented with *p*-coumaric acid

Cell (**a**) and medium (**b**) fractions were collected by centrifugation after galactose induction in the presence of 1.0 mM of *p*-coumaric acid. All chromatograms are shown on a comparative scale, except for standards.

Supplementary Table 1. Primers used in this work

Primer name	Sequence (5' – 3')
AcPT1_5'UTR_Fw	TTTTCTTCAGGGTTGAATTCTTG
AcPT1_3'UTR_Rv	ATGTCGGGTTGTTCTTCACC
AcPT1_qRTPCR_Fw	CCATGGAGGATCCGCATTAC
AcPT1_qRTPCR_Rv	ATTCCATGCATCCGATCTCC
Ac26SrRNA_Fw	GGTGCGAGTTCTATCGGGTA
Ac26SrRNA_Rv	CACTTGGAGCTCTCGATTCC
AcPT1TP_Fw	CACCATGGGCGTCTCTAACAGTGG
AcPT1TP_Rv	AAATTGCGCAGAGACATTAT
AcPT1_TOPO_Fw	CACCATGGCGTCTCTAACAGTGGG
AcPT1_TOPO_Rv	TCAGCGCACAAGCGCGATAA
ΔNtHMGCoAR_Fw	AAGCGGCCGCATGACTGCAGACCAATTGGTG
ΔNtHMGCoAR_Rv	GCTTAATTAATTAGGATTTAATGCAGGTGACG
CPR_Fw	AAAAAACCCCGGATCATGCAATCATCAAGCAGCTC
CPR_Rv	ACCAAGCTTACTCGATTACCATACATCACGCAGAT
ΔTP-AcPT1_Sc_EcoRI_Fw	GCGAATTCATGCAGAGAAGAGGTTACAA
ΔTP-AcPT1_Sc_SacI_Rv	CGGAGCTCTCATCTAACCAAAGCAATCA
AcPT1_EcoRI_Fw	AAGAATTCATGGCGTCTCTAACAGTGG
AcPT1_SacI_Rv	AAGAGCTCTCAGCGCACAAGCGC
ΔTP-AcPT1_EcoRI_Fw	AAGAATTCATGCAACGAAGAGGTTATAAAAATCATT

Supplementary Table 2. Primary metabolism-related PTs used in phylogenetic analysis

PT member	Plant species	Protein ID
**Tocopherol biosynthesis**		
AtVTE2-1	*Arabidopsis thaliana*	NP_849984.1
GmVTE2-1	*Glycine max*	NP_001241496.1
HvVTE2-1	*Hordeum vulgare*	BAJ97902.1
LaVTE2-1	*Lactuca sativa*	ACN78585.1
OsVTE2-1	*Oryza sativa*	XP_015644510.1
TaVTE2-1	*Triticum aestivum*	ABB70123.1
ZmVTE2-1	*Zea mays*	ACG45339.1
**Plastoquinone biosynthesis**		
AtVTE2-2	*Arabidopsis thaliana*	NP_001078138
GmVTE2-2	*Glycine max*	NP_001237900
HaVTE2-2	*Helianthus annuus*	XP_022000593.1
OsVTE2-2	*Oryza sativa*	XP_015646905.1
ZmVTE2-2	*Zea mays*	NP_001146703.1
**Phylloquinone biosynthesis**		
AtABC4	*Arabidopsis thaliana*	NP_001117518.1
GmABC4	*Glycine max*	XP_003532605.1
HaABC4	*Helianthus annuus*	XP_021989185.1
OsABC4	*Oryza sativa*	NP_001049226.1
ZmABC4	*Zea mays*	NP_001152170.1
**Chrolophyll biosynthesis**		
AtATG4	*Arabidopsis thaliana*	NP_190750.1
GmATG4	*Glycine max*	NP_001239633.1
HaATG4	*Helianthus annuus*	XP_021970771.1
OsATG4	*Oryza sativa*	ABO31092.1
ZmATG4	*Zea mays*	NP_001142204.1
**Heam *a* biosynthesis**		
AtCOX10	*Arabidopsis thaliana*	NP_566019.1
GmCOX10	*Glycine max*	XP_003556552.1
HaCOX10	*Helianthus annuus*	XP_022025736.1
OsCOX10	*Oryza sativa*	EEC70799.1
ZmCOX10	*Zea mays*	AFW89544.1
**Ubiquinone biosynthesis**		
AtPPT1	*Arabidopsis thaliana*	NP_567688
GmPPT	*Glycine max*	XP_006602724.1
HaPPT	*Helianthus annuus*	XP_021993218.1
OsPPT1	*Oryza sativa*	BAE96574.1
ZmPPT	*Zea mays*	NP_001148558.1

Supplementary Table 3. Specialized metabolism-related PTs used in phylogenetic analysis

PT member	Plant species	Protein ID
**Asteraceae**		
AcPT1	*Artemisia capillaris*	LC425153
**Polaceae**		
HvHGGT	*Hordeum vulgare*	AAP43911.1
OsHGGT	*Oryza sativa*	AAP43913.1
TaHGGT	*Triticum aestivum*	AAP43912.1
ZmHGGT	*Zea mays*	XP_008659772.1
**Rutaceae**		
ClPT1	*Citrus limon*	BAP27988.1
**Apiaceae**		
PcPT	*Petroselinum crispum*	BAO31627.1
PsPT1	*Pastinaca sativa*	AJW31563.1
PsPT2	*Pastinaca sativa*	AJW31564.1
**Fabaceae**		
AhR3'DT-1	*Arachis hypogaea*	AQM74173.1
AhR3'DT-2	*Arachis hypogaea*	AQM74174.1
AhR3'DT-3	*Arachis hypogaea*	AQM74175.1
AhR3'DT-4	*Arachis hypogaea*	AQM74176.1
AhR4DT-1	*Arachis hypogaea*	AQM74172.1
GmC4DT	*Glycine max*	BAW32575.1
GmG2DT	*Glycine max*	BAW32578.1
GmG4DT	*Glycine max*	NP_001235990
GmIDT1	*Glycine max*	BAW32576.1
GmIDT2	*Glycine max*	BAW32577.1
GuA6DT	*Glycyrrhiza uralensis*	AIT11912.1
GuILDT	*Glycyrrhiza uralensis*	AMR58303.1
LaPT1	*Lupinus albus*	AER35706.1
LjG6DT	*Lotus japonicus*	ARV85585.1
SfFPT	*Sophora flavescens*	AHA36633.1
SfG6DT	*Sophora flavescens*	BAK52291.1
SfiLDT	*Sophora flavescens*	BAK52290.1
SfN8DT-1	*Sophora flavescens*	BAG12671.1
SfN8DT-2	*Sophora flavescens*	BAG12673.1
SfN8DT-3	*Sophora flavescens*	BAK52289.1

Supplementary Table 3. Specialized metabolism-related PTs used in phylogenetic analysis - *continued*

PT member	Plant species	Protein ID
**Moraceae**		
CtIDT	*Cudrania tricuspidata*	AJD80983.1
MaIDT	*Morus alba*	AJD80982.1
**Cannabaceae**		
HlPT-1	*Humulus lupulus*	BAJ61049.1
HlPT-2	*Humulus lupulus*	AJD80255.1
**Hypericaceae**		
HcPT	*Hypericum calycinum*	ALD84371
**Boraginaceae**		
AePGT	*Arnebia euchroma*	ABD59796.2
AePGT4	*Arnebia euchroma*	ANC67957.1
AePGT6	*Arnebia euchroma*	ANC67959.1
LePGT1	*Lithospermum erythrorhizon*	BAB84122.1
LePGT2	*Lithospermum erythrorhizon*	BAB84123.1
