# Supplementary material for: Isolation of Artemisia capillaris membrane-bound di-prenyltransferase for phenylpropanoids and redesign of artepillin C in yeast
Source: Commun Biol. 2019 Oct 18;2:384. doi: 10.1038/s42003-019-0630-0 (PMC6802118; doi:10.1038/s42003-019-0630-0)
Supplement: Supplementary file 2 — Descriptions of Additional Supplementary Files [file 42003_2019_630_MOESM2_ESM.docx]

**Description of additional supplementary files**

Supplementary Data 1: Raw data for Figures 2a, 2b, 3c, 3d, 4b, 4c, 5b, and 5c.
