# Supplementary material for: Isolation of Artemisia capillaris membrane-bound di-prenyltransferase for phenylpropanoids and redesign of artepillin C in yeast
Source: Commun Biol. 2019 Oct 18;2:384. doi: 10.1038/s42003-019-0630-0 (PMC6802118; doi:10.1038/s42003-019-0630-0)
Supplement: Supplementary file 5 — Reporting Summary [file 42003_2019_630_MOESM5_ESM.pdf]

nature research

Corresponding author(s): Kazufumi Yazaki

Last updated by author(s): Sep 4, 2019

Reporting Summary

Nature Research wishes to improve the reproducibility of the work that we publish. This form provides structure for consistency and transparency in reporting. For further information on Nature Research policies, see Authors & Referees and the Editorial Policy Checklist.

Statistics

For all statistical analyses, confirm that the following items are present in the figure legend, table legend, main text, or Methods section.

n/a	Confirmed	
☐	☒	The exact sample size (*n*) for each experimental group/condition, given as a discrete number and unit of measurement
☐	☒	A statement on whether measurements were taken from distinct samples or whether the same sample was measured repeatedly
☐	☒	The statistical test(s) used AND whether they are one- or two-sided<br>*Only common tests should be described solely by name; describe more complex techniques in the Methods section.*
☒	☐	A description of all covariates tested
☒	☐	A description of any assumptions or corrections, such as tests of normality and adjustment for multiple comparisons
☐	☒	A full description of the statistical parameters including central tendency (e.g. means) or other basic estimates (e.g. regression coefficient) AND variation (e.g. standard deviation) or associated estimates of uncertainty (e.g. confidence intervals)
☐	☒	For null hypothesis testing, the test statistic (e.g. *F*, *t*, *r*) with confidence intervals, effect sizes, degrees of freedom and *P* value noted<br>*Give P values as exact values whenever suitable.*
☒	☐	For Bayesian analysis, information on the choice of priors and Markov chain Monte Carlo settings
☒	☐	For hierarchical and complex designs, identification of the appropriate level for tests and full reporting of outcomes
☒	☐	Estimates of effect sizes (e.g. Cohen's *d*, Pearson's *r*), indicating how they were calculated

Our web collection on statistics for biologists contains articles on many of the points above.

Software and code

Policy information about availability of computer code

Data collection

RNA-seq library was obtained with illumina Hiseq2000 100bp Paiered-End, Next Generation Sequencing, FASTQ Data output.
Nucletide and protein sequences were collected from NCBI database.
qRT-PCR data were obtained with Bio-Rad CFX Manager.
UPLC/MS, HPLC/MS and HPLC data were obtained with LabSolutions (Shimadzu), LC solution (Shimadzu), and D-2000 Elite (Hitachi), respectively.
Microscopic images were taken with VB-7000 Ver. 1.20 (Keyence), FV31S-SW (Olympus).

Data analysis

RNA-seq data were analyzed using CLC GenomicsWorkbench 5.5.2 CLC Bio, QIAGEN).
Amino acid identity and local blast analyses were done with Bioedit.
Phylogenetic analysis was done with MEGA6.
Transmembrane regions and transit peptides of proteins were predicted with TMHMM Server, v. 2.0 and ChloroP 1.1 Server, respectively.
Kinetic data were analyzed with Sigmaplot 12.
Microscope images were analyzed with Adobe Photoshop CS3 and FV31S-SW (Olympus).
Figures from quantification data were made by Microsoft Excel 2016.
Statistic analysis was done by R software ver. 3.4.1

For manuscripts utilizing custom algorithms or software that are central to the research but not yet described in published literature, software must be made available to editors/reviewers. We strongly encourage code deposition in a community repository (e.g. GitHub). See the Nature Research guidelines for submitting code & software for further information.

Data

Policy information about availability of data

All manuscripts must include a data availability statement. This statement should provide the following information, where applicable:

- Accession codes, unique identifiers, or web links for publicly available datasets
- A list of figures that have associated raw data
- A description of any restrictions on data availability

The nucleotide sequences of genes reported in this work have been deposited in NCBI (accessions LC425153 and LC425154). The raw RNA-seq reads have been submitted to the DDBJ (accession DRA007286). The source datasets underling main figure 2a, 2b, 3c, 3d, 4a, 4b, 4c, 5b, and 5c are available as a Source Data file.

Field-specific reporting

Please select the one below that is the best fit for your research. If you are not sure, read the appropriate sections before making your selection.

☒ Life sciences ☐ Behavioural & social sciences ☐ Ecological, evolutionary & environmental sciences

For a reference copy of the document with all sections, see nature.com/documents/nr-reporting-summary-flat.pdf

Life sciences study design

All studies must disclose on these points even when the disclosure is negative.

Sample size	No sample size calculation was performed in advance. Samples sizes are determined according to comparable experiments in previously articles.
Data exclusions	In qRT-PCR analysis (Figure 2a), the expression value of one stem replicate was excluded because of a clear technical mistake in pipetting. The other data were not excluded.
Replication	In vitro enzymatic assays were performed with three independent incubations. The other quantification datasets were obtained in use of at least three biological replicates.
Randomization	No experiments were carried out in randomized conditions because randomization was not seriously relevant in this study.
Blinding	No investigations were blinded because blinding was not seriously relevant to our non-clinical experiments.

Reporting for specific materials, systems and methods

We require information from authors about some types of materials, experimental systems and methods used in many studies. Here, indicate whether each material, system or method listed is relevant to your study. If you are not sure if a list item applies to your research, read the appropriate section before selecting a response.

Materials & experimental systems

n/a	Involved in the study	
☒	☐	Antibodies
☐	☒	Eukaryotic cell lines
☒	☐	Palaeontology
☒	☐	Animals and other organisms
☒	☐	Human research participants
☒	☐	Clinical data

Methods

n/a	Involved in the study	
☒	☐	ChIP-seq
☒	☐	Flow cytometry
☒	☐	MRI-based neuroimaging

Eukaryotic cell lines

Policy information about cell lines

Cell line source(s)	Saccharomyces cerevisiae lines were used in this study. COUM11, a p-coumaric acid producing line, and DD104, a terpenoid-engineered line, were kindly provided by Dr. F. Ververidis (Technological Educational Institute of Crete), and Dr. M. J. C. Fischer (INRA, Colmar) and Dr. D. Werck-Reichhart (CNRS, University of Strasbourg), respectively. Δpdr5 and Δsnq2 strains were kindly provided by Dr. T. Miyakawa (Hiroshima University). AD12345678 strain was kindly provided by Dr. A. Goffeau (Université Catholique de Louvain). DAC strains were created to produce drupanin and artepillin C using the DD104 strain as a host in this study.
Authentication	Each yeast line is authenticated by auxotrophic phenotyping and the productivity of relevant molecules. DAC strains were

Authentication	also validated by PCR for introduced plasmids.
Mycoplasma contamination	Not tested.
Commonly misidentified lines (See ICLAC register)	n/a
